# Efficacy of an Adenoviral Vectored Multivalent Centralized Influenza Vaccine

**DOI:** 10.1038/s41598-017-14891-y

**Published:** 2017-11-02

**Authors:** Amy Lingel, Brianna L. Bullard, Eric A. Weaver

**Affiliations:** School of Biological Sciences, Nebraska Center for Virology, University of Nebraska, Lincoln, USA

## Abstract

Mice were immunized with Adenovirus expressing the H1-con, H2-con, H3-con and H5-con HA consensus genes in combination (multivalent) and compared to mice immunized with the traditional 2010–2011 FluZone and FluMist seasonal vaccines. Immunized mice were challenged with 10–100 MLD_50_ of H1N1, H3N1, H3N2 and H5N1 influenza viruses. The traditional vaccines induced robust levels of HA inhibition (HI) titers, but failed to protect against five different heterologous lethal influenza challenges. Conversely, the multivalent consensus vaccine (1 × 10^10^ virus particles (vp)/mouse) induced protective HI titers of ≥40 against 8 of 10 influenza viruses that represent a wide degree of divergence within the HA subtypes and protected 100% of mice from 8 of 9 lethal heterologous influenza virus challenges. The vaccine protection was dose dependent, in general, and a dose as low as 5 × 10^7^ vp/mouse still provided 100% survival against 7 of 9 lethal heterologous influenza challenges. These data indicate that very low doses of Adenovirus-vectored consensus vaccines induce superior levels of immunity against a wide divergence of influenza subtypes as compared to traditional vaccines. These doses are scalable and translatable to humans and may provide the foundation for complete and long-lasting anti-influenza immunity.

## Introduction

Using methods previously published, the CDC estimates that influenza vaccination prevented 1.9 million influenza illnesses and 67,000 influenza-associated hospitalizations during the 2015-16 flu season^1^. However, there were 40 million influenza illnesses and 970,000 influenza-associated hospitalizations in that same year^2^. To put this in other terms our current influenza vaccine programs and technologies reduce influenza infections and hospitalizations by 4.75% and 6.9%, respectively. Society is becoming more aware of the weakness of the “flu shot” and more skeptical of the vaccine. Antigenic mismatches, limited stocks, and, most recently, the CDC recommended against using FluMist due to reports that it was completely ineffective^3^. There is no doubt that there is a need for more effective vaccine technologies.

In 2008, a novel strain of influenza started in Mexico and spread to Texas and California later that year. At the time the vaccine was a complete mismatch to this novel “Swine Flu” virus. There was 20.5% amino acid divergence between the vaccine and the circulating influenza hemagglutinins (HA)^4^. By the end of the pandemic 24% of the global population had been infected with the 2009 Swine influenza^5^. There were ~68 million influenza infections and 275,304 influenza-associated hospitalizations in the USA alone^6^. The 2009 swine flu pandemic was a cautionary reminder of our vulnerability to infectious diseases like influenza. Although we invest significant efforts into global monitoring, antiviral drug discovery and advanced vaccine technology, the pandemic was unstoppable.

In addition to the 1918 H1N1 pandemic, the Asian Flu pandemic of 1957–1958 was a result of H2N2 influenza virus. Another global pandemic, the Hong Kong Flu of 1968–1969, was caused by H3N2 influenza virus^7,8^. In 1997 there were 18 individual incidents of avian to human transmission of H5N1^9^. Between 2003 and 2016, 856 human cases of avian H5N1 influenza and 452 deaths were reported to the World Health Organization (WHO)^10^. Recently, scientists have shown that H5N1 can be adapted to confer transmission between ferrets through aerosols^11,12^. These studies highlight the potential of H5N1 to mutate to a highly transmissible human-to-human strain with a high mortality. H7N9, H7N2, and H9N2 have also been found to sporadically infect humans, usually due to close contact with infected birds^8,13^.

Vaccination against seasonal influenza is key to reducing the burden of infection. However, a recent study on influenza vaccine efficacy concluded that our current trivalent inactivated (FluZone) and live-attenuated cold-adapted (FluMist) vaccines are only 59% effective with variable results in different age groups^14^. In addition, it is very difficult to predict which influenza strains will be circulating in the upcoming years. Recent advances in influenza vaccine technology include the use of high-dose vaccines for the elderly and the inclusion of an additional B virus in the Quadrivalent influenza vaccine formulation^15,16^. These advances will help increase the overall efficacy of the influenza vaccine as well as increase the likelihood of influenza B virus vaccine coverage. However, the potential for a vaccine mismatch and failure is still a very real possibility.

An ideal influenza vaccine would be inexpensive, provide long-lasting immunity, require few immunizations, and would work against all variants of the virus. Researchers have explored several approaches to improve on our current vaccine technology. The ectodomain of the M2 matrix protein is highly conserved amongst influenza viruses and represents an attractive target for creating a cross-reactive vaccine antigen^17^. While this approach has shown some promise it appears that complete protection is limited. Other researchers have focused on driving immunity towards the stalk region of the hemagglutinin. Since the stalk region is more conserved than the globular head, it is thought that highly effective cross-reactive neutralizing antibodies directed towards this region would provide universal protection against heterologous influenza challenge^18–20^. Challenge studies have shown this approach to be effective, but data are limited to only one or two heterologous influenza challenge strains. Another approach is to use the conserved internal genes of influenza as vaccine antigens. Recent studies using bacterially expressed or MVA expressed proteins such as NP, M1, and PB1 have shown to be protective against an influenza virus challenge^21,22^. Consensus genes were initially pioneered as broadly-reactive HIV vaccine antigens and shown to provide greater levels of cross-protective immunity than wildtype antigens^23–25^. Consequently, scientists have applied this approach to the influenza hemagglutinin (HA) gene^4,26–28^. A vaccine that is matched to the challenge virus will always induce the greatest levels of immunity. However, in the case of vaccine mismatch the consensus vaccine genes tend to show greater levels of cross-protective immunity. While all of these approaches show some levels of protection against heterologous challenge there is a lack of uniformity in the vaccine doses, challenge viruses and varying challenge doses that make it impossible to compare the vaccine platforms. Our approach utilizes a dose dependent-study on the vaccines, includes multiple heterologous influenza strains for each subtype, and involves a highly stringent 10–100 MLD_50_ influenza virus challenge.

In this study we have applied the centralized gene vaccine approach to four human relevant influenza virus HA genes, H1, H2, H3, and H5. The centralized genes were incorporated into replication-defective Adenovirus (Ad) viral vector systems and tested in combination (multivalent) to protect against a wide panel of divergent H1, H3 and H5 influenza isolates. In addition, we compared our Ad-vectored centralized vaccines to the traditional FluZone and FluMist vaccine platforms. We found that *in vitro* and *in vivo* analyses indicate that the Ad-vectored centralized vaccines can provide high levels of protection against a wide array of divergent influenza strains at very low doses. The protection conferred by the Ad-vectored centralized consensus genes was superior to that induced by traditional vaccine technology. We feel that combining the robust gene delivery of Ad vaccines with the use of centralized HA genes may provide a foundation of immunity that is capable of providing high levels of cross-protective immunity against many, if not all human influenza strains.

## Results

### Phylogenetic Analyses of Centralized HA genes

**Figure 1:**
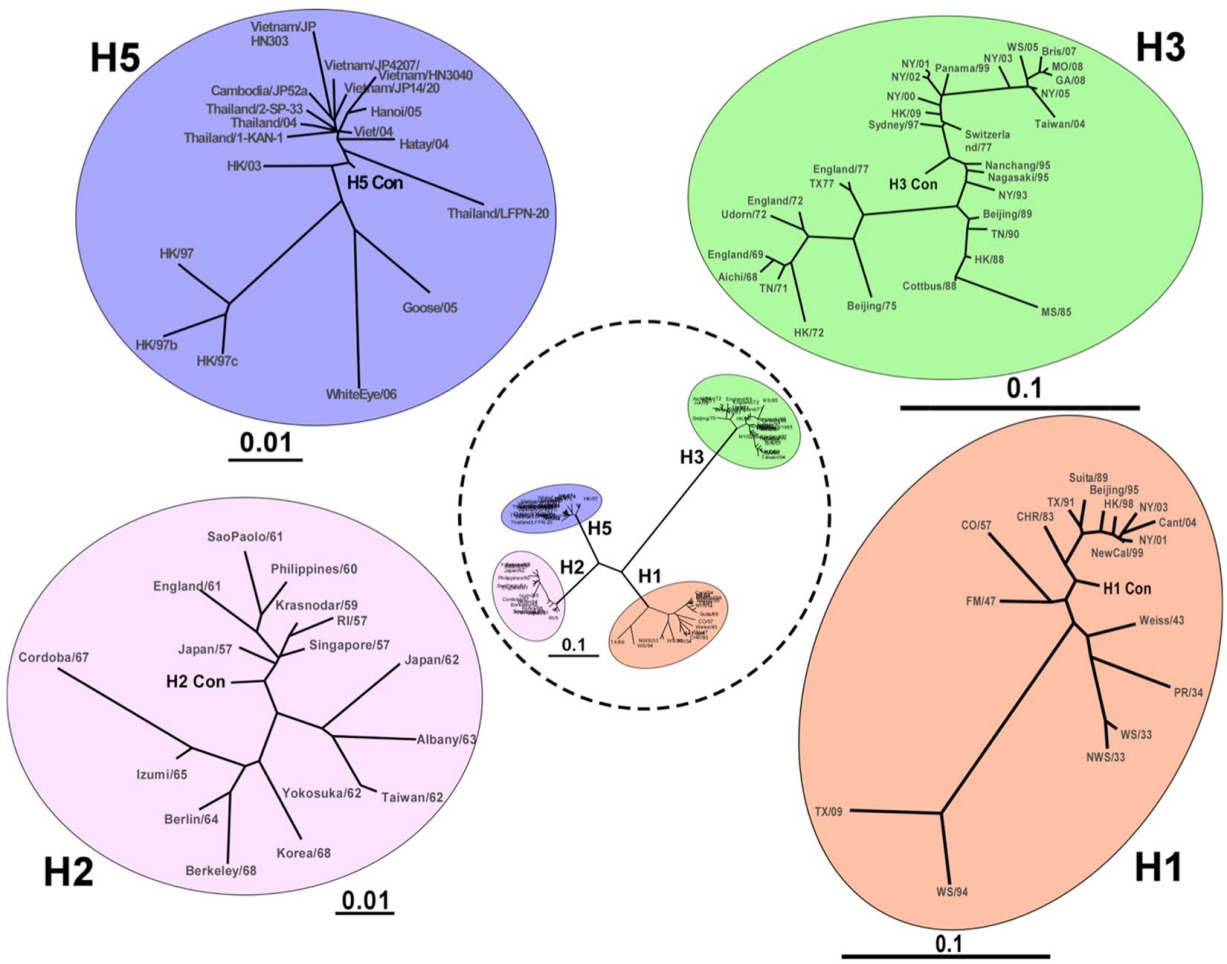
Phylogenetic Analyses of Centralized Hemagglutinin Genes. The genetic relationships between the consensus genes and wildtype genes are shown in unrooted neighbor-joining phylogenetic trees. H1, H2, H3 and H5 consensus genes are shown localizing to the center of the tree. Black bars indicate the percent divergence of the HA amino acid sequences. The central tree (dotted line) illustrates the degree of genetic diversity between the HA subtypes.

The relationship of the consensus genes to other wildtype genes is shown in Fig. 1. All of the consensus genes localize to the central region of each of the respective trees. The average genetic distance of the H1-con, H2-con, H3-con and H5-con consensus genes to the wildtype genes was 7.04%, 2.86%, 5.44% and 1.65%, respectively. However the maximum genetic distance between wildtype H1-con, H2-con, H3-con and H5-con genes was 20.7%, 7.9%, 14.7% and 5.1%, respectively. The centralized genes act to reduce genetic diversity to mismatched genes by being equidistant to all analyzed wildtype genes (Fig. 1). The amount of genetic diversity within each subtype is variable with the highest rates of diversity in the H1 and H3 strains. The least divergent influenza subtype is H5, which could be attributed to limited infections in humans or its comparatively shorter period of circulation. Avian H5N1 strains were analyzed separately and not used to create the centralized H5 consensus gene. Avian H5 strains, particularly low pathogenic avian influenza (LPAI) strains have considerably higher rates of genetic diversity that are >14.0%. This may be the result of the continued evolution of LPAI stains in wild birds. The center tree shows the significant divergence between the influenza HA subtypes. The evolution of influenza allows for extreme levels of plasticity in the HA genes and the HA subtypes can be up to 60% divergent and still have identical functionality (Fig. 1).

### Efficacy of FluMist and FluZone Vaccination

**Figure 2:**
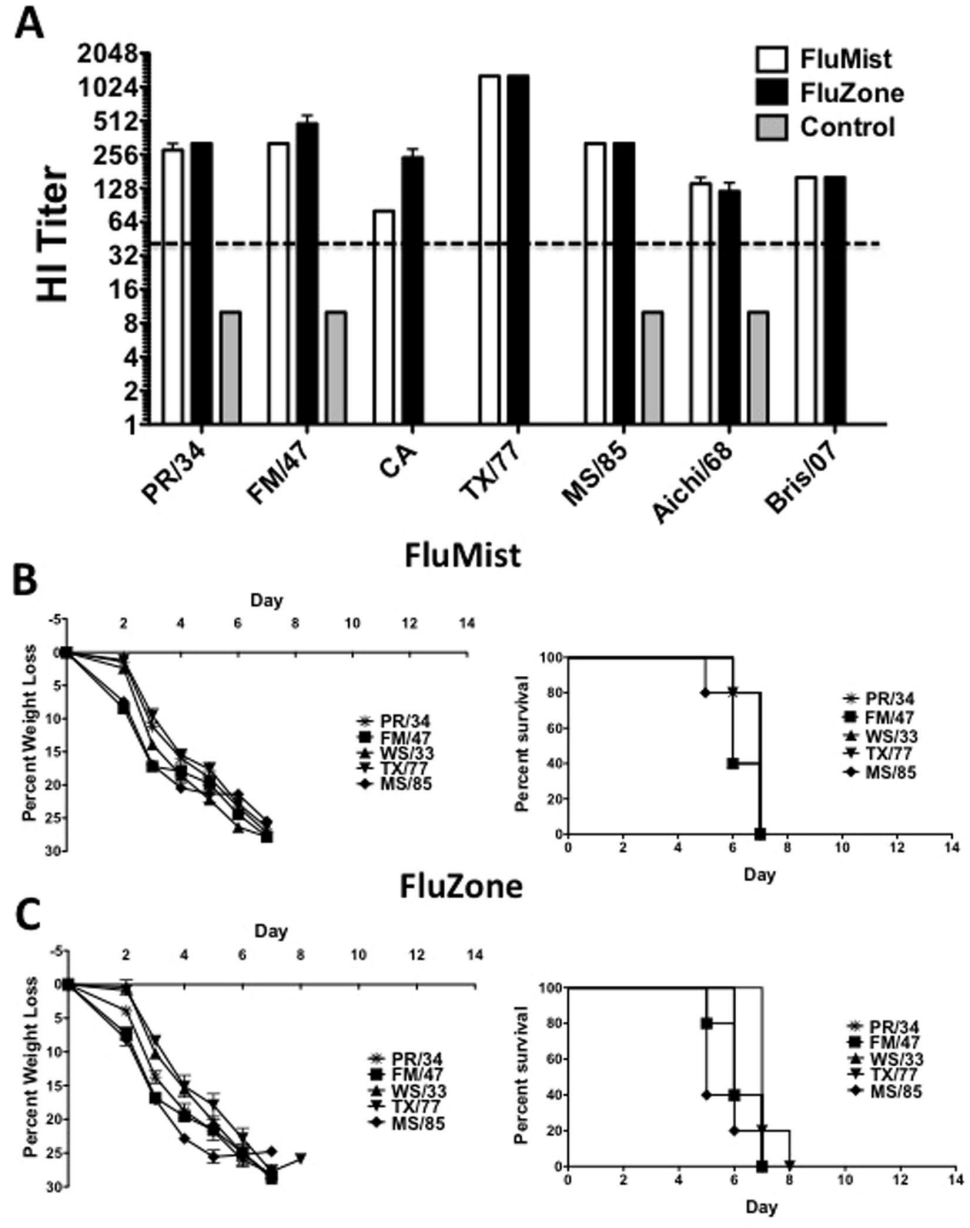
Protection Against Lethal H1N1, H3N1 and H3N2 Influenza Virus by Traditional Trivalent Vaccines. Mice were immunized and boosted with the 2010–2011 FDA approved traditional vaccines. The vaccines consisted of either the live-attenuated FluMist or the killed-inactivated FluZone containing influenza A/California/07/2009 X-179A (H1N1)pdm09, A/Victoria/210/2009 X-187 (an A/Perth/16/2009-like virus) (H3N2) and B/Brisbane/60/2008. The mice were immunized using the same vaccine schedule as the Ad vectored vaccines. Sera collected 2 weeks post-boosting was assayed for HI antibody titers against a panel of H1N1, H3N1 and H3N2 influenza viruses (**A**). Sera from unimmunized control mice were also analyzed for non-specific HI antibody titers. The dotted line represents a theoretical titer indicative of protection (HI titer of 40). The vaccinated mice were challenged with lethal doses of mouse-adapted influenza viruses 4 weeks post-boosting. The mice were monitored for weight loss, disease and survival (**B**). Mice that lost ≥25% of their baseline weight were humanely euthanized. Weight loss and survival for unvaccinated control groups are shown in Figs 3 and 4.

Mice were primed and boosted with two traditional influenza vaccines, FluMist and FluZone. Sera collected after boosting showed significant levels of anti-influenza HI titers against a broad array of divergent H1 and H3 influenza strains (Fig. 2A). In general, a HI titer of 40 is considered protective and correlates to a 50% reduction in influenza infection^29^. The HI titers against the CA/09 strain were easily above 40 in mice vaccinated with either traditional vaccine. In general, similar levels of HI titers were induced by both traditional vaccine platforms. However, the FluZone vaccine induced statistically higher anti-CA/09 influenza antibody titers (p =<0.05). Sera from control mice were included in order to determine non-specific anti-influenza background. There were no detectable HI titers in these mice and a base HI titer of 10 was assigned to these sera. The vaccinated mice were challenged with 3 divergent H1 and 2 divergent H3 mouse-adapted lethal influenza viruses. Even though the HI titers indicate that the mice have specific anti-influenza antibodies, none of the FluMist vaccinated mice were protected against any of the lethal influenza challenges (Fig. 2B). All of the mice had similar disease and weight loss profiles as compared to the control unvaccinated mice. Similarly, there were no differences in protection in the FluZone immunized mice against any of the influenza strains (Fig. 2C). None of the mice immunized with the traditional vaccine platforms survived any of the lethal influenza challenges.

### HI Titers After Multivalent Consensus HA Vaccination

**Figure 3:**
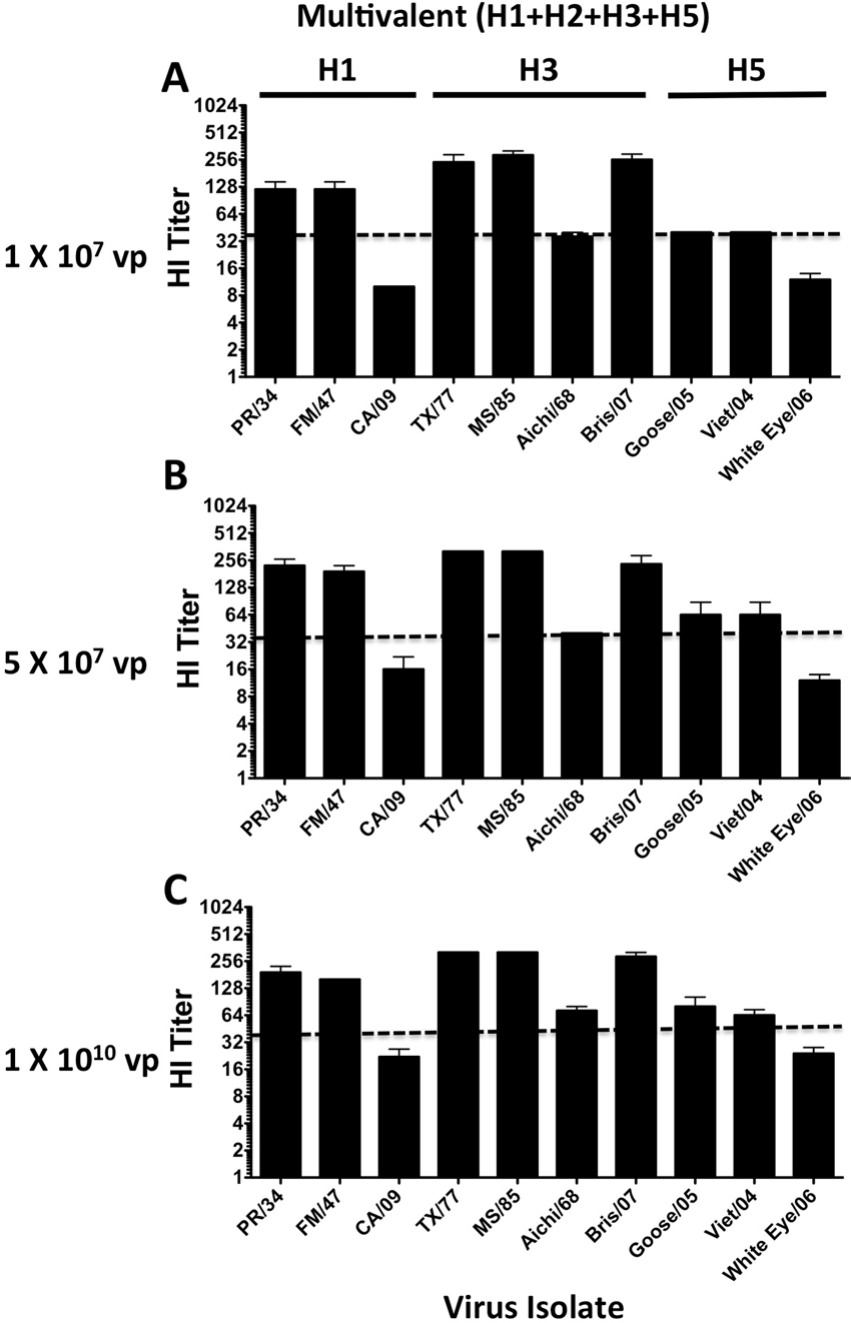
Hemagglutination Inhibition Assays after Multivalent HA Vaccination. Mice were immunized and boosted with a multivalent mixture of Ad4 and Ad5 expressing the H1, H2, H3 and H5 consensus HA genes at various doses. Sera from the vaccinated mice were assayed for HI antibody titers against a wide panel of divergent influenza strains. The HI titers induced by the lowest dose of vaccine, 1 × 10^7^ vp/mouse are shown (**A**). A low dose vaccine of 5 × 10^7^ vp/mouse is shown (**B**). A high dose vaccine of 1 × 10^10^ vp/mouse is shown (**C**). The dotted line represents a theoretical titer indicative of protection.

In order to determine if the centralized vaccine genes could be used to create a multivalent H1, H3 and H5 vaccine, we immunized groups of mice with a combination of Ad vaccines expressing all four HA con genes individually. The immunized mouse sera were assayed for HI antibody titers against 10 divergent influenza viruses representing H1N1, H3N1, H3N2 and H5N1 influenza viruses (Fig. 3). As seen previously, the HI titers increased in a dose-dependent manner^4^. The lowest dose of 1 × 10^7^ vp/mouse induced HI titers above 40 for 8 out of 10 influenza strains (Fig. 3A). Increasing the dose to 5 × 10^7^ vp/mouse slightly increased the HI titers. However, HI titers above 40 were obtained against only 8 out of 10 influenza strains (Fig. 3B). Increasing the vaccine dose to 1 × 10^10^ vp/mouse also increased the overall HI titers. However, again only 8 out of 10 strains reached a titer of 40 (Fig. 3C). The CA/09 and the White Eye/06 appear to be considerably more difficult to inhibit as compared to the other influenza strains. Not surprisingly, these strains were the most divergent from the centralized H1-con and H5-con genes as compared to the other wildtype strains (Fig. 1).

### Protection Against Divergent Lethal H1N1 Influenza Challenge After Multivalent Consensus HA Vaccination

**Figure 4:**
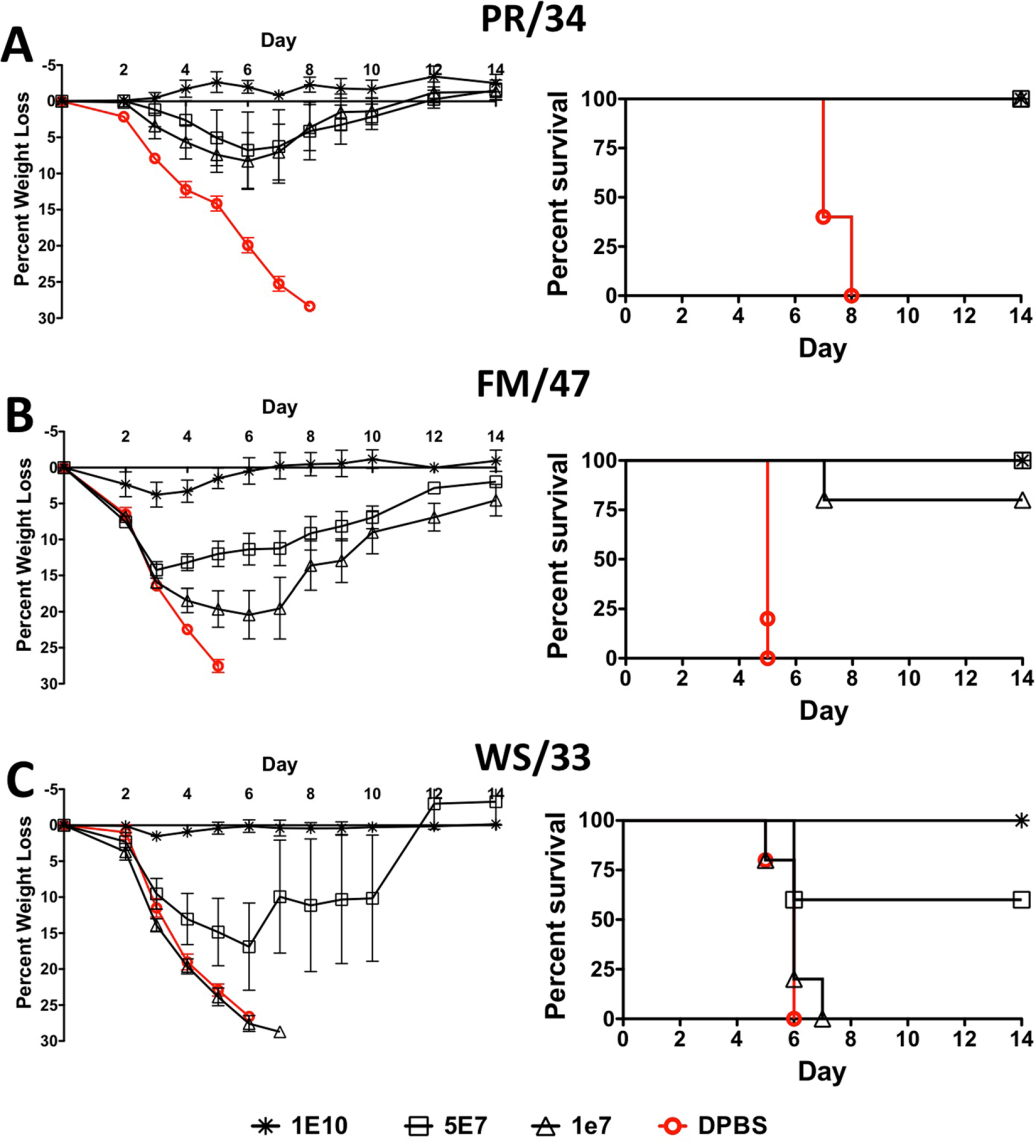
Protection Against Lethal H1N1 Influenza Virus Using a Multivalent Centralized H1, H2, H3 and H5 Vaccine. Mice that were vaccinated with various doses of the multivalent vaccine expressing all four consensus HA genes were challenged with 100 MLD_50_ of mouse-adapted influenza A/Puerto Rico/8/34 (**A**), A/Fort Monmouth/1/47 (**B**), or A/WS/33 (**C**). The mice were monitored for weight loss, disease and survival. Mice that lost ≥25% of their baseline weight were humanely euthanized.

The multivalent Ad vaccine protected 100% of mice from death due to a lethal influenza PR/34 challenge at all vaccine doses (Fig. 4A). However, only the highest dose was capable of completely preventing weight loss and disease. The highest and lower dose of 5 × 10^7^ vp/mouse protected 100% of mice against death by a lethal influenza FM/47 challenge (Fig. 4B). The lowest dose protected 80% of the mice against death. Again, only the highest dose completely prevented disease, weight loss and death. Only the highest dose of multivalent vaccine was capable of inducing 100% protection of mice against weight loss, disease and death when challenged with a lethal dose of WS/33 influenza virus (Fig. 4C). The lower vaccine dose of 5 × 10^7^ vp/mouse resulted in 60% survival, whereas, 0% of the mice vaccinated with 1 × 10^7^ vp/mouse survived the WS/33 influenza challenge. Here we show that the vaccine is protecting in a dose-dependent manner against H1N1 influenza virus challenges. The genetic distance between the centralized consensus H1-con gene and the PR/34, FM/47 and WS/33 was 8.5%, 4.8% and 8.0%, respectively. Therefore, genetic distance was not the only defining characteristic of protection. Otherwise the greatest levels of protection would have been against FM/47 and not PR/34.

### Protection Against Divergent Lethal H3N1 and H3N2 Influenza Challenge After Multivalent Consensus HA Vaccination

**Figure 5:**
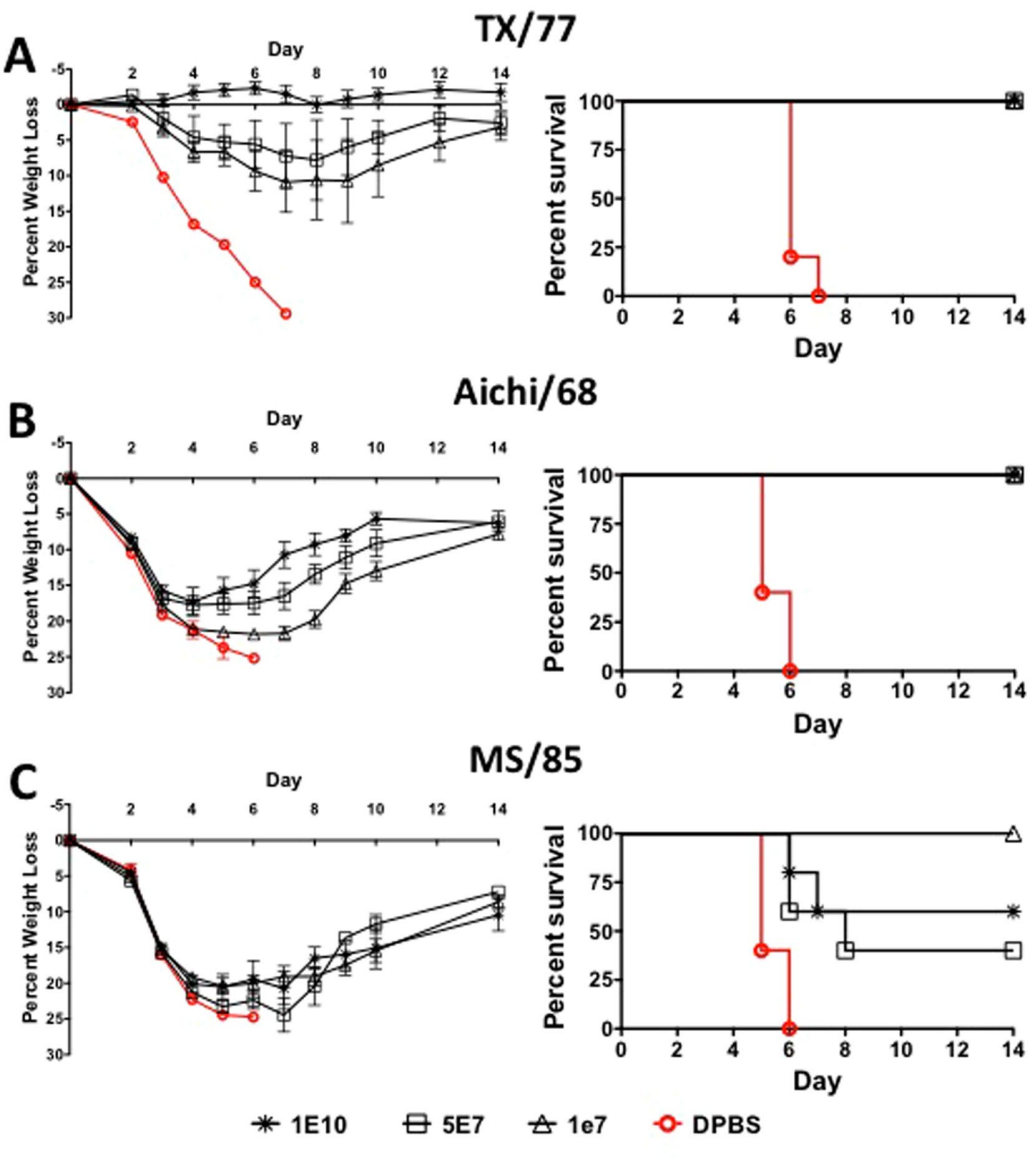
Protection Against Lethal H3N1 and H3N2 Influenza Virus Using a Multivalent Centralized H1, H2, H3 and H5 Vaccine. Mice that were vaccinated with various doses of the multivalent vaccine expressing all four consensus HA genes were challenged with 100 MLD_50_ of mouse-adapted influenza A/Texas/1/77 (**A**), 10 MLD_50_ of A/Aichi/2/68 and 10 MLD_50_ of A/Mississippi/1/85 (**B** and **C**, respectively). The mice were monitored for weight loss, disease and survival. Mice that lost ≥25% of their baseline weight were humanely euthanized.

Mice vaccinated with the multivalent Ad vaccine were protected against lethal H3N1 and H3N2 influenza challenges. Mice vaccinated with the multivalent vaccine were 100% protected against death at all doses when challenged with influenza TX/77 and Aichi/68 (Fig. 5A and B). The highest dose of vaccine prevented weight loss and disease in the TX/77 challenged mice (Fig. 5A). However, the lower doses of vaccine were associated with weight loss and disease. Although 100% of the mice were protected against death from a lethal challenge of Aichi/68, all of the mice at all vaccine doses showed signs of disease and weight loss (Fig. 5B). All vaccinated mice showed signs of disease and weight loss at all vaccine doses when challenged with MS/85 lethal influenza virus (Fig. 5C). Interestingly, the lowest dose of vaccine, 1 × 10^7^ vp/mouse, protected mice from death when challenged with MS/85. Vaccine doses of 5 × 10^7^ and 1 × 10^10^ vp/mouse resulted in 40% and 60% survival, respectively. The mice were protected against the TX/77 and Aichi/68 strains in a dose-dependent manner. However, there was no dose-dependent effect against the MS/85 challenged mice. It is possible that this phenomenon is the result of interference by immunodominant epitopes on the other genes at higher doses. The genetic distance between the centralized consensus H3-con gene and the TX/77, Aichi/68 and MS/85 strains was 6.1%, 9.0% and 7.6%, respectively. In this case the greatest level of protection was observed in the mice challenged with the least divergent strain. However, the vaccine efficacy was better against the Aichi/68 strain as compared to the MS/85 strain even though the Aichi/68 had the highest level of genetic divergence of all strains tested.

### Protection Against Divergent Lethal H5N1 Influenza Challenge After Multivalent Consensus HA Vaccination

**Figure 6:**
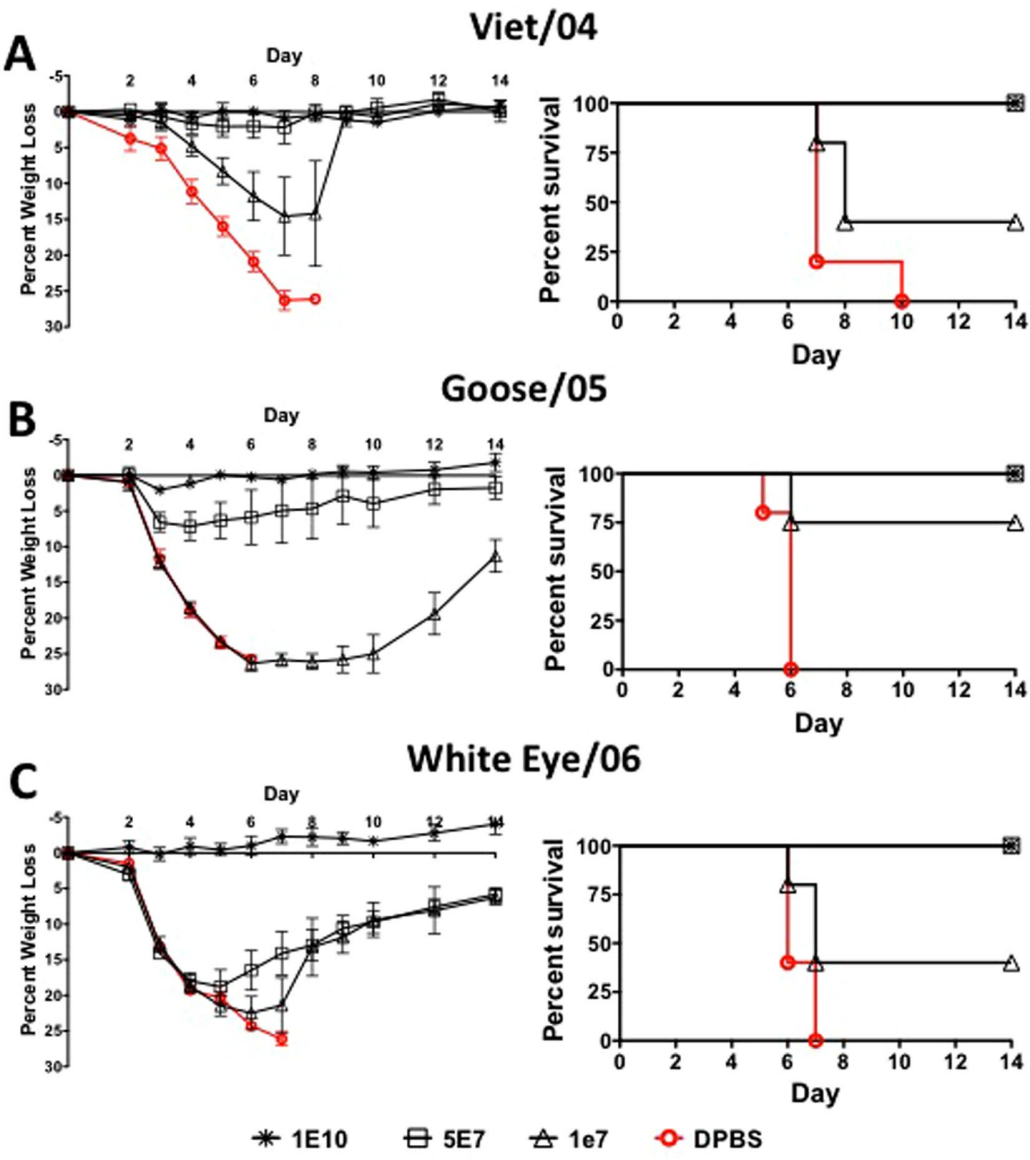
Protection Against Lethal H5N1 Influenza Virus Using a Multivalent Centralized H1, H2, H3 and H5 Vaccine. Mice that were vaccinated with various doses of the multivalent vaccine expressing all four consensus HA genes were challenged with 100 MLD_50_ of mouse-adapted influenza A/Vietnam/1203/04 (**A**), A/BH Goose/Qinghai/A/05 (**B**) and A/Japanese White Eye/Hong Kong/1038/2006 (**C**). The mice were monitored for weight loss, disease and survival. Mice that lost ≥ 25% of their baseline weight were humanely euthanized.

The multivalent Ad vaccine protected mice against lethal H5N1 influenza challenges. The 5 × 10^7^ and 1 × 10^10^ vp/mouse doses of vaccine completely protected mice against a lethal Viet/04 influenza challenge (Fig. 6A). These doses of vaccine resulted in the absence of weight loss, disease and death. The lowest vaccine dose of 1 × 10^7^ vp/mouse only protected 40% of mice from death with this lethal challenge. The 1 × 10^10^ vp/mouse vaccine dose also completely protected mice from the Goose/05 and White Eye/06 influenza challenges (Fig. 6B and C). Mice immunized with the middle dose, 5 × 10^7^ vp/mouse, showed signs of weight loss and disease, but were 100% protected from death. The lowest dose of vaccine, 1 × 10^7^ vp/mouse, resulted in more severe weight loss and disease and only protected 40%, 75% and 40% of mice from death against Viet/04, Goose/05 and White Eye/06 influenza challenges, respectively. The genetic distance between the centralized consensus H5-con gene and the Viet/04, Goose/05 and White Eye/06 was 0.7%, 2.6% and 3.4%, respectively. Again the vaccine worked in a dose-dependent manner for all virus challenges and the efficacy correlates with genetic distance. Where the least divergent had the highest levels of protection and the most divergent had the lowest level of protection.

### Neutralization and T Cell Immunity

**Figure 7:**
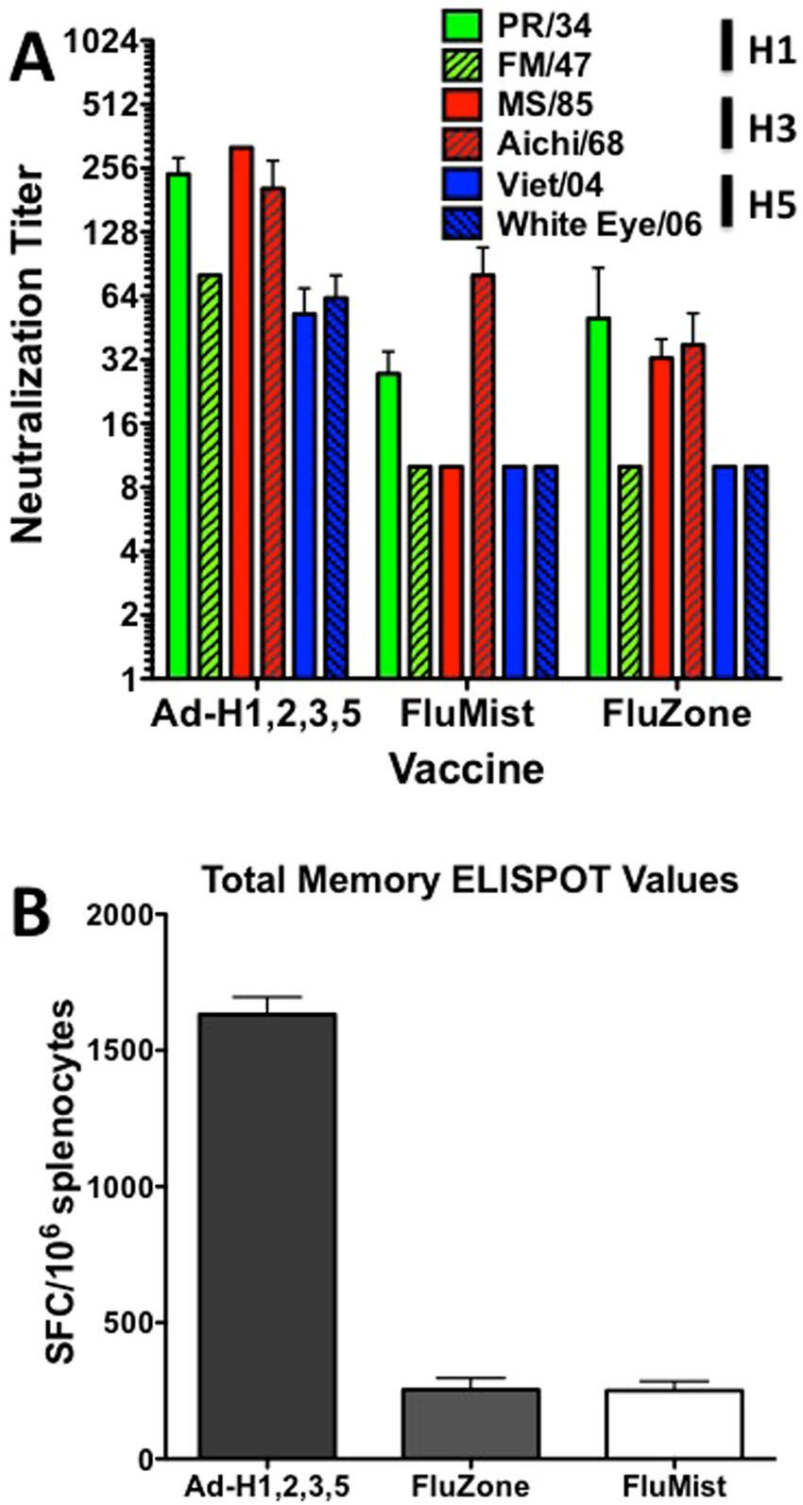
Micro-neutralization and Memory T Cell. Sera from vaccinated mice was serially diluted and incubated with 50 TCID_50_ of each indicated influenza virus for 1 h. 2.5 × 10^5^ MDCK cells were added to the wells and the plates were incubated for 5 days. 50 µl of 0.5% chicken RBC solution was added to all wells and incubated at room temperature for 1 h before reading. The neutralization titer was determined as the highest dilution of sera to prevent agglutination (**A**). Splenocytes from the vaccinated mice were collected and pooled. The splenocytes were stimulated with peptides representing the 4 H3 proteins, H3-con, TX/77, Aichi/68 and MS/85. The peptides consisted of 17-mers overlapping by 12 amino acids. The assays were run in duplicate. Interferon gamma secreting cells were detected using AN18 and R4-6A2 antibodies and the total spot forming cells (SFC)/10^6^ splenocytes are shown (**B**).

HI titers have been a standard for determining vaccine efficacy. However, they lack the ability to determine anti-influenza functionality. Therefore we looked at the ability of immunized mouse sera to neutralize H1, H3 and H5 influenza strains (Fig. 7A). Clearly the multivalent Ad vaccinated mice induced significantly greater neutralization titers against all of the influenza strains tested as compared to the FluZone and FluMist vaccinated mice. In addition, only the multivalent Ad vaccine immunized mice were capable of neutralizing the H5 strains as expected since the FluMist and FluZone vaccines do not contain any H5 immunogen. The default titer was set at 10 since this was the starting dilution. Figure 7B shows the overall anti-H3 memory T cell responses in the vaccinated mice. The Ad vaccine clearly induced greater levels of anti-H3 T cell immunity as compared to the FluZone and FluMist vaccines (Fig. 7B). It was surprising, that there were any detectable levels of anti-H3 T cells in the FluZone immunized mice. However, the doses used are 10-fold greater than a human equivalent and the booster is given at 4 weeks post-priming. These T cell data may represent a product of cross-presentation of vaccine antigen.

### Ad Vaccine Infectivity and HA Protein Expression

**Figure 8:**
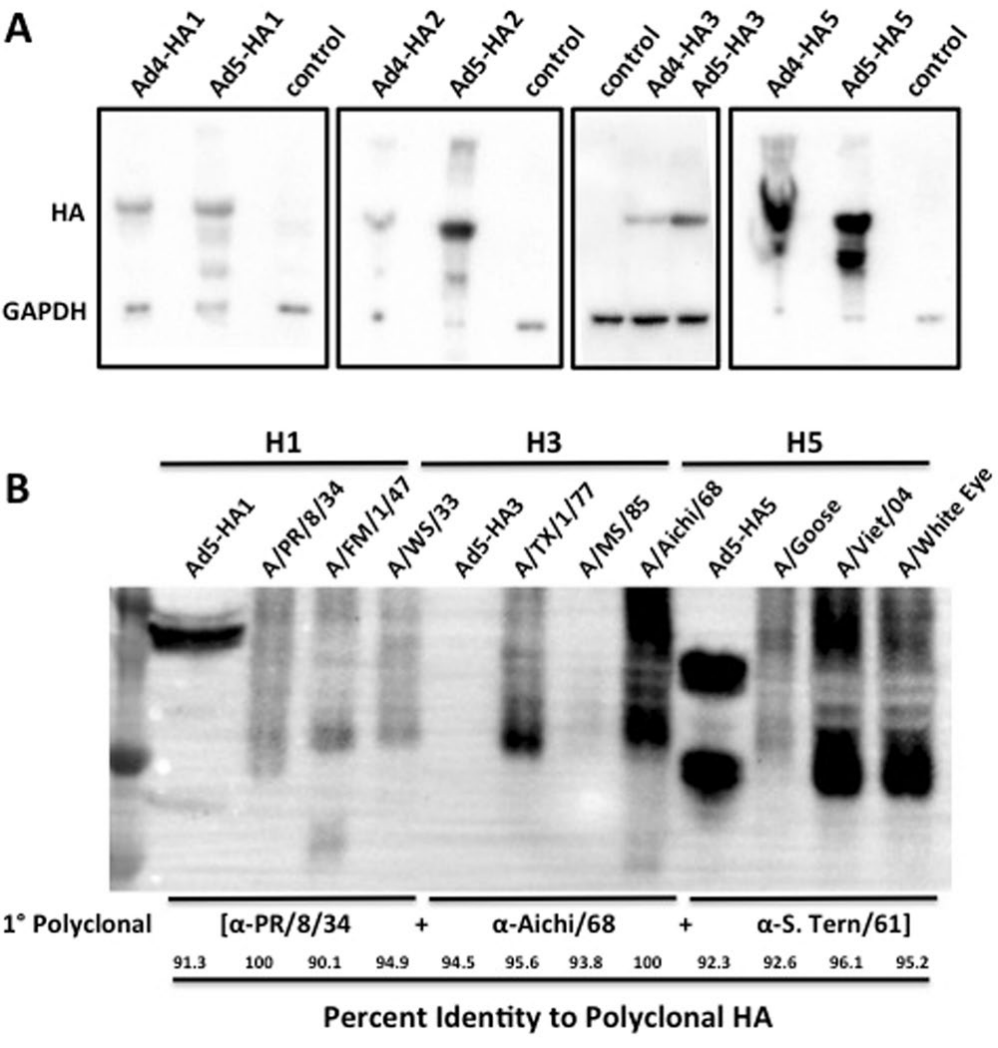
Western Blot for Consensus HA Expression. 293 cells were infected with all 8 of the Ad vaccine vectors individually. The cells were harvested after 24 hrs. and cell lysates were run on a 8% SDS-PAGE gel. Protein was transferred to PVDF membranes and detected using polyclonal goat antiserum. Goats were immunized with A/PR/8/34, A/Singapore/1/57, A/Aichi/2/68, and A/Tern/S. Africa/61 for H1, H2, H3 and H5 protein expression, respectively. Due to differences in polyclonal anti-HA affinities the exposure times were different for each of the protein blots. A GAPDH control was also used as a standard. In order to determine strain-specific detection of HA proteins using polyclonal sera, the consensus proteins and wildtype virus proteins were probed with a combination of anti-H1, H3 and H5 goat polyclonal sera. The polyclonal antibody was detected by α-Goat-HRP secondary antibody. The blot was developed and imaged as one figure (**B**). The percent identity of the virus used to create the polyclonal sera relative to the antigen being detected is shown.

Table 1 shows the relative virus particles/ml in each CsCl Ad prep as determined by OD260. We determined virus infectivity using 2 assays, the TCID_50_ and AdenoX Rapid titer kit. When we analyzed the Ad5-HA-con vaccines with the AdenoX Rapid titer kit we determined that the Ad-HA-con vaccines had a typical Ad5 virus particle (vp)/infectious unit (iu) of 30, 47, 143 and 190 for Ad-H1, Ad-H2, Ad-H3 and Ad-H5, respectively. Similar results for Ad5 were obtained using the TCID_50_ assay. However, we were unable to detect Ad4 infectious particles using the AdenoX Rapid titer kit. In addition, the TCID_50_ values for Ad4 indicate a considerably high vp/iu ratio. However, it was observed that at a lower dilution it took longer for Ad4 plaques to form than the Ad5 viruses. This indicates that the 293 cell may not be the best cell line for titering Ad4 virus stock and therefore, the infectivity appears lower. Since, Ad4 is a species E adenovirus, the complementing Ad5-E1 in 293 cells may not be optimal for virus growth. The Ad-HA vaccines were used to infect 293 cells and the HA protein expression was determined by western blot (Fig. 8A). All 8 Ad vaccines expressed the HA proteins as detected by polyclonal serum. We included a GAPDH control as a reference. The HA3 protein expression appears to be significantly reduced as compared to the other Ad-HA vaccines as indicated by the strong GAPDH signal as compared to the HA signal. All consensus HA genes were cloned with the exact same restriction enzymes, identically codon-optimized, contained the same Kozak’s sequence and stop codons. The specific reason for the decreased H3 protein expression remains unclear. However, one possibility is that the HA3 protein appears reduced because of ineffective cross-reactivity by the primary polyclonal antibody as compared to the other consensus HA proteins. In order to determine the cross-reactivity we tested the ability of the polyclonal anti-H1, H3 and H5 antibodies to detect consensus and wildtype influenza proteins (Fig. 8B). The consensus HA proteins and their wildtype influenza virus counterparts were separated by SDS-PAGE and blotted on PVDF membrane in parallel. The blots were then probed with a combination of the polyclonal sera. The percent identity between the HA used to create the polyclonal antibody and the wildtype and consensus proteins is shown (Fig. 8B). The polyclonal anti-H1 and H5 sera were capable of detecting the consensus proteins and the wildtype proteins. However, the anti-H3 polyclonal detected the homologous Aichi/68 virus and partially cross-reacted with TX/77. The H3 consensus and MS/85 HA proteins were not detected under the same conditions (Fig. 8B). The MS/85 virus stock has a known viral titer as determined by HA assay. Therefore, the lack of MS/85 detection correlates well with our hypothesis that H3-con protein expression levels may not be reduced, but that detection is limited by the strain-specific polyclonal antibody.

**Table 1:** Titer and Infectivity of Viral Vector Vaccines.

	Ad4-H1	Ad4-H2	Ad4-H3	Ad4-H5	Ad5-H1	Ad5-H2	Ad5-H3	Ad5-H5
**Viral Particle (OD260)/ml**	3.50E + 12	4.70E + 12	1.40E + 12	2.90E + 12	2.60E + 12	6.68E + 11	1.16E + 12	1.48E + 12
**AdenoX Rapid Titer/ml**	ND	ND	ND	ND	8.47E + 10	1.41E + 10	8.10E + 09	7.80E + 09
**TCID**_**50**_ **/ml**	2.28E + 09	3.16E + 11	1.78E + 07	1.08E + 08	6.62E + 10	3.16E + 11	3.16E + 11	1.37E + 12

### Linear Antibody Epitope Analyses

**Figure 9:**
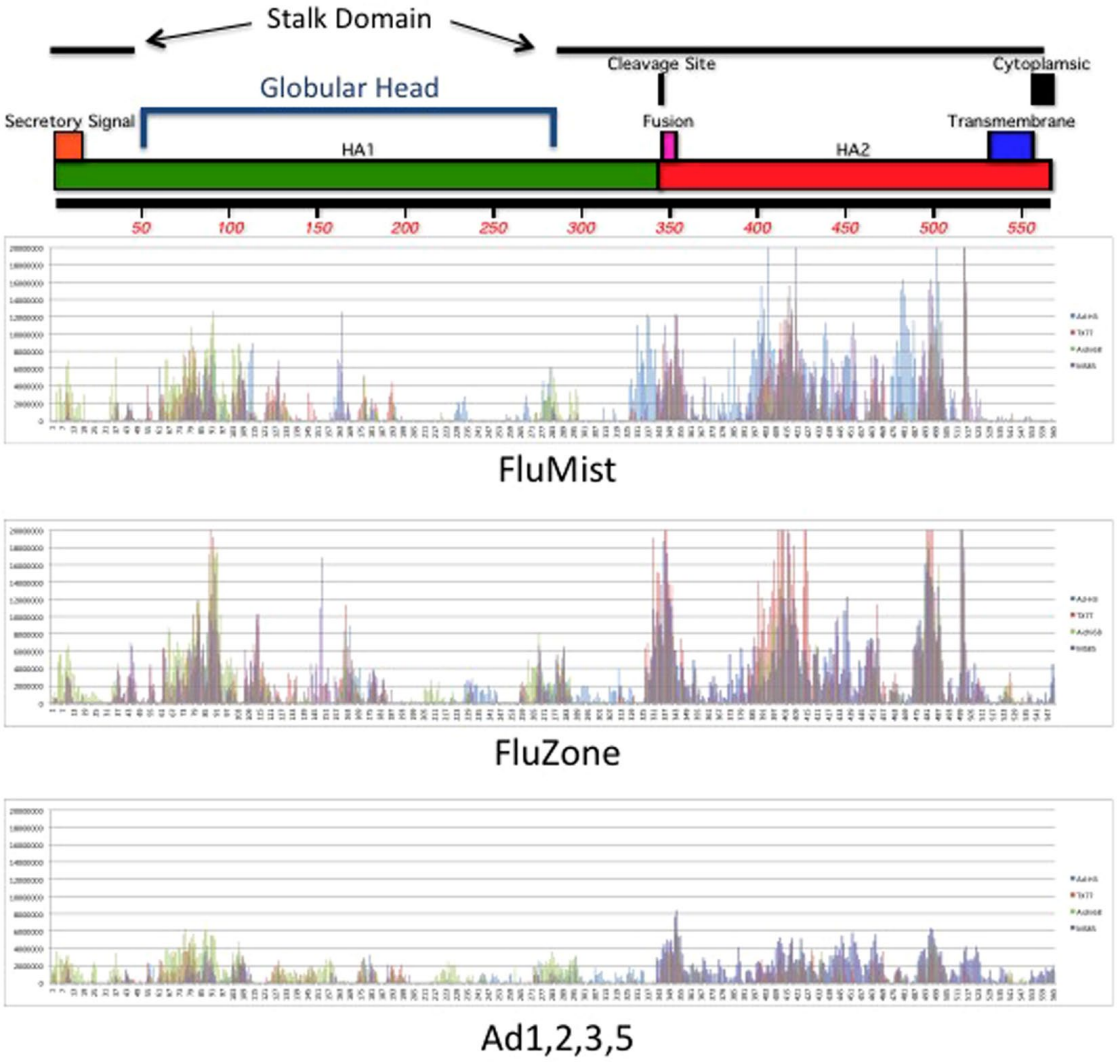
Linear B Cell Epitope Analysis. Sera from vaccinated mice were pooled and used to probe a peptide microarray that consisted of 2080 peptides that were linked to the slide in duplicate. The peptides were 15-mers and overlapped by 14 amino acids. The anti-H3 influenza antibodies were detected using Cy3 goat anti-mouse IgG and read using an Axon GenePix 4000B scanner. Antibodies against the H3-con, TX/77, Aichi/68 and MS/85 peptides are indicated by the blue, red, green, and purple bars, respectively.

The ability of each vaccine to induce antibodies that recognize linear H3 epitopes was analyzed using a peptide microarray. Groups of 5 mice were vaccinated and boosted with the Ad multivalent vaccine (using the 1 × 10^10^ dose), FluMist and FluZone. We pooled the sera from the immunized animals and used it to probe the peptide microarray. The peptide microarray contained peptides representing the HA from all three H3 influenza strains and the H3-con protein (Fig. 9). There were no obvious differences in the antibody binding profiles in regard to the location of signal. However, the traditional FluMist and FluZone vaccines induced higher peptide binding signal than the Ad vaccine. Peptide binding antibody was found across the HA protein that included both stalk and globular head antibodies (Fig. 9). Antigen-specific antibody binding for the H3-con, TX/77, Aichi/68 and MS85 HA proteins are indicated by the blue, red, green and purple bars, respectively.

## Discussion

Centralized vaccine antigens have been studied for their ability to induce cross-protective immunity for more than a decade. Indeed a vaccine antigen that is genetically equidistant to all known variants is more likely to induce broader cross-reactive immunity than any wildtype antigen. So, when the vaccine does not match the contemporary strains, a centralized gene would likely induce a superior level of cross-protective immunity. Phylogenetic analyses of our 4 consensus HA genes shows that they do localize to the center of the tree and are more genetically equidistant to all other strains as compared to the wildtype isolates (Fig. 1). The consensus genes were incorporated into Adenovirus types 4 and 5 for evaluation as influenza vaccine vectors. In a previous study, we showed that a H1-con antigen was immunogenic and did induce greater levels of cross-protective immunity as compared to wildtype HA antigens^28^. In a subsequent paper we showed that the H3-con and H5-con antigens were also immunogenic and could provide cross-protective immunity against multiple heterologous lethal influenza challenges when delivered individually^4^. While our study focuses on more human relevant influenza subtypes, others have applied this approach to avian influenza viruses with potential for zoonotic transmission such as avian H5N1, H7N9 and H9N2^26,30,31^. Unfortunately, we are unable to test if the H2-con gene induced any protection at this time. However, the data from the H1, H3 and H5 consensus genes support the likelihood that a H2-con vaccine would induce some significant level of protection. In order to determine if the vaccine antigens would interfere with each other and reduce the vaccine efficacy, we delivered the centralized consensus antigens in combination as a multivalent vaccine and then assessed the protection by HI titers and heterologous lethal influenza challenges. In addition we compared our consensus Ad-vectored vaccine to two traditional influenza vaccine platforms, FluMist and FluZone.

We found that mice vaccinated with Adenovirus vectored consensus vaccine doses of 1 × 10^10^ and 5 × 10^7^ vp/mouse induced very similar levels of protection whether delivered individually or in combination, with one exception. When delivered individually, the H3-con gene completely protected mice from weight loss, disease and death at the highest dose when challenged with a lethal dose of influenza MS/85. Protection with the H3-con gene was dose-dependent and, as expected, the lower doses of vaccine were less capable of protecting the mice against influenza MS/85^4^. However, when the consensus genes were delivered together in the multivalent vaccine, only the lowest dose of vaccine resulted in 100% survival (Fig. 5C). Interestingly, the HI titers are consistently high against the MS/85 influenza isolate when delivered as a monovalent or multivalent vaccine at all doses (Figs 2 and 4). The simplest explanation for the differences in protection against this isolate may be that there is interference between the immune responses induced by the multivalent consensus vaccine. Immune bias in the high dose vaccine may have circumvented the MS/85 strain-specific immunity that was induced at the lower dose. It is possible that, when in combination, there is interference by an immunodominant epitope that reduces the strain-specific protection against the MS/85 influenza virus. An analysis of the immunophenotype of mice vaccinated with high and low doses of the multivalent vaccine may elucidate the mechanism for the inverted dose response by this vaccine against this strain.

Overall, the highest dose of vaccine protected 100% of mice against 8 of 9 lethal challenges when delivered as a multivalent vaccine. Considering that the challenge doses are 10–100 MLD_50_ and representative of extremely high mortality rates, the results of protection at the highest vaccine dose may better resemble a real world influenza pandemic scenario. Mice showed 100% survival at a vaccine dose of 5 × 10^7^ vp/mouse against 7 out of 9 divergent lethal strains. This dose is very translatable to humans and indicates just how effective the vaccine is against extremely pathogenic influenza viruses. The 5 × 10^7^ vp/mouse vaccine dose did not provide 100% survival against two viruses, WS/33 and MS/85; however, they still resulted in 60% and 40% survival, respectively. Initially, we though that the lack of H3-con efficacy could be attributed to reduced protein expression levels as detected by western blot (Fig. 8). Although the western blots are not quantitative, the ratio of HA protein/GAPDH protein is qualitatively low compared to the other HA-con proteins. Interestingly, a subsequent study that tested the polyclonal sera to detect wildtype influenza virus proteins showed that while the H1 and H5 polyclonal sera detected the consensus HA proteins and all of their wildtype counterparts; the anti-H3 polyclonal antibody was not capable of detecting the H3-con or the MS/85 influenza virus proteins under these same conditions (Fig. 8B). When the H3-con protein was probed alone it was detected using the anti-H3 polyclonal serum, but at much longer exposure parameters than the other HA proteins. The percent identity between the virus and the antigen used to make the polyclonal does not always correlate with the detection of most similar proteins although, in this case, the homologous Aichi/68 was best detected followed by the next most identical TX/77 virus. Based on body weight, the dose of 5 × 10^7^ vp/mouse equates to ~1.5 × 10^11^ vp/human. These doses have been used in human clinical trials and found to be safe with little toxic side effects^32–35^. Studies that evaluated the vaccines in the context of less stringent influenza challenges would most likely show an increase in vaccine efficacy at even lower doses of vaccine.

Mice that were vaccinated with the highest dose of the multivalent vaccine did not show any signs of disease when challenged with any of the 6 heterologous H1N1 or H5N1 viruses. However, the highest dose of vaccine was only able to completely protect mice against the TX/77 H3N1 strain. High dose vaccinated mice did show signs of disease and weight loss when challenged with Aichi/68 and MS/85 H3N2 influenza viruses. These two strains required much more virus to reach lethal levels. Therefore, the inoculum was much greater than all other challenge strains and even with the higher dose we were only able to reach a dose of 10 MLD_50_. Therefore, it is possible that neutralizing antibodies are present but are depleted due to the overall viral antigen load in the virus challenge. If we were to improve the lethality of these viruses by mouse-adaptation using sequential passages in mice, we could reduce the required overall amount of viral particles to achieve a lethal dose. Consequently, the bulk of the intranasal virus challenge may be neutralized upon delivery. In every challenge tested there was a vaccine dose that provided 100% survival against a lethal challenge. However, these mice do show signs of disease and weight loss, but fully recover from the infection. Therefore, we can assume that anti-influenza cellular immunity and variations in the cytokine responses due to the vaccination and subsequent virus challenge may be playing a significant role in the protective effects. In fact, Fig. 7B shows significantly higher overall T cell responses in the Ad vaccinated mice. Future studies directed at understanding the T cell dynamics and cytokine/chemokine profiles may be informative as to the details of this mechanism of protection.

Surprisingly, the traditional vaccines induced HI titers that were similar or greater than the Ad-vectored vaccines. However, none of the FluZone or FluMist vaccinated mice were capable of surviving protection when challenged with a divergent lethal influenza strain, whereas, mice vaccinated with the Ad-vectored consensus vaccine were capable of providing complete protection in most cases. We then characterized the functionality of the FluZone, FluMist and Ad vaccine antibodies using a neutralization assay. We found that the neutralizing capacity of the antibodies induced by the traditional vaccine were significantly lower than that induced by the Ad multivalent vaccine. Indicating that while there is substantial anti-influenza antibody, it is not necessarily functional (Fig. 7A). In addition, we screened the induced immune responses to recognize H3 linear epitopes using a peptide microarray (Fig. 9). Again, we found robust antibody responses against both the globular head and stalk domains. In fact, the fluZone and FluMist vaccinated mice showed a greater intensity of antibody binding as compared to the Ad multivalent vaccinated mice. We were unable to identify any significant linear epitopes that could explain the lack of neutralization induced by the traditional vaccine. Perhaps, the neutralizing epitopes are conformational and not detected with the microarray. Figure 7B indicates that there is some cross-priming of cellular immunity by the inactivated FluZone vaccine, however, these responses are quite limited. The live-attenuated FluMist vaccine also shows some cellular immunity which is expected since this is a live attenuated virus. Again, these T cell responses are low as compared to the Ad vaccinated mice. In general, the T cell responses induced by FluMist is limited and confined primarily to unvaccinated children^36^. Similar studies using the 2011–2012 FluMist did show some level of heterologous protection against a lethal A/PR/8/34 influenza challenge^37,38^. However, these studies were done using vaccine doses 15-fold higher than in our studies. Subsequently, the heterologous vaccine efficacy was attributed to cross-reactive T cell immunity. Our data demonstrate the importance of high levels of intracellular expression of the vaccine transgenes, resulting cellular immunity and, possibly, the adjuvanting effects of the Ad-vector itself^39^. The inability of the traditional influenza vaccines to provide protection against a lethal virus challenge illustrates how strikingly powerful the Ad-HA vaccines are inducing anti-influenza immunity.

This study clearly shows the efficacy of a consensus gene based Ad-vectored vaccine. Traditional influenza vaccine efficacy is questionable and the inevitability of vaccine mismatch is always present. Our vaccine induces broad levels of protection against a wide array of divergent lethal influenza challenges that are far superior to that of either traditional influenza vaccine platform. While we do not dismiss the utility of the traditional vaccine platform, the incorporation of centralized influenza genes may act to reinforce a foundation of immunity against a wider variety of influenza isolates than can be accomplished using current vaccine technology. Since the formulation of a centralized HA vaccine would not change from year to year they could be incorporated into the FluZone or FluMist platforms to be used alongside the strain-specific vaccine formulation. Repeated administration of the centralized vaccine in the annual vaccine formulation would also act to boost the cross-protective immune responses and, perhaps, increase the affinity of shared T and B cell epitopes. Alternatively, the consensus genes could be administered in the context of an Ad-vectored vaccine. Pre-existing immunity to Ad-vectored vaccines is a principal concern and deservingly so. However, if used in naïve infants, an Ad-vectored consensus influenza vaccine could establish a broad foundation of anti-influenza immunity. In addition, the naïve infants would also be immune to the type-specific Adenoviruses used as the vaccine vector. Alternatively, one could use low seroprevalent Adenoviruses that have been proven to be effective influenza vaccine vectors^38^. Influenza viruses are continuously evolving through genetic drift. This is a major obstacle in creating a universal vaccine. Including the consensus antigens in the annual vaccine formulation would likely induce a selective pressure to evolve and thus escape the immunity induced by the consensus antigens. Therefore, virus surveillance and monitoring would still be critical to controlling outbreaks. In addition, as more viruses are sequenced and the databases are updated, new consensus antigens could be derived in order to account for the new virus variants. This unique vaccine strategy provides a foundation for anti-influenza immunity that may provide broader cross-protective immunity against heterologous challenges and future antigens would require continuous monitoring and modification.

The magnitude of protection in this study is significantly understated. Here we show that an Ad-vectored prime/boost vaccine that contains 4 centralized consensus vaccine antigens can provide 100% survival against all 9 heterologous lethal influenza challenges. It has to be noted that the majority of the challenges are using a lethal dose of 100 MLD_50_. To put it another way, there is enough virus in these influenza challenges to kill 50 mice and represents a 100% mortality rate. To our knowledge this is the first “two-shot” vaccine that has ever been evaluated using multiple vaccine doses and 9 lethal heterologous influenza challenges. Clearly, the protection at these extremes demonstrates that this vaccine could be very effective if the challenge virus was similar to a natural endemic influenza infection. It is very possible that a vaccine similar to the one described in this study could provide complete and life-long protection against all known human influenza variants.

## Materials and Methods

### Centralized Gene Construction and Phylogenetic Analyses

The centralized consensus HA genes were created as previously described^4,28^. In brief, sequences that represent the major branches of the phylogenetic tree for each subtype were selected. The representative sequences are aligned using ClustalX and the most common amino acid at each position is chosen as the consensus sequence. The alignments and analyses indicate that these centralized genes retain critical functional domains such as the secretory signal, polybasic connecting peptide, cleavage and fusion sites, transmembrane domains and cytoplasmic tail. The consensus Hemagglutinins named H1-con, H2-con, H3-con, and H5-con were 566, 562, 566 and 568 amino acids in length, respectively. Unrooted neighbor-joining phylogenetic trees were created using ClustalX v2.1 and viewed using TreeView v1.6.6 (Fig. 1). For safety concerns, the polybasic cleavage site in the H5-con gene was reduced to the single arginine cleavage site that is found in low pathogenic avian influenza virus. The HA genes were codon-optimized for mammalian expression and synthesized by Genscript. Inc.

### Adenovirus Vaccines

In order to create a prime/boost immunization strategy, we used two different species of Human Adenoviruses. We cloned all four HA-con genes into the species C Adenovirus type 5 replication-defective E1/E3 deleted Ad-Easy vector system as previously described^4^. Briefly, the HA-con genes were cloned into the pShuttle-CMV plasmid using BglII and SalI restriction enzymes. The shuttle plasmid was linearized with PmeI and cotransformed with pAd-Easy into BJ5183 cells. Kanamycin resistant colonies were analyzed for recombination. The Ad5 recombinants were confirmed by restriction digestion and midiprepped using the Qiagen Hi-Speed Midiprep kit. This was repeated for all HA-con genes. The recombinant plasmids were digested with PacI and transfected into 293 cells using Polyfect. Plaques were observed and the virus was amplified using subsequent freeze/thaw passages. The Ad5 vaccine constructs are diagramed in Supplemental Fig. 1. All four HA-con genes were also cloned in place of the E1 genes of the species E Adenovirus type 4 as previously described^4^. The strategy for cloning the HA-con genes into Ad4 has been diagramed in Supplemental Fig. 2. Briefly, the CMV-HA-con PolyA expression cassette was PCR amplified from the Ad-Easy system and fused to Frt-Zeo-Frt using overlapping PCR that included AscI sites on the 5′ and 3′ ends. The AscI flanked expression and Zeo cassette was cloned into an Ad4-E1 shuttle plasmid. The pAd-E1-HA-con shuttle plasmids were linearized with PacI and recombined into the pAd4-gDNA plasmid by cotransformation in BJ5183 cells. Kanamycin and Zeocin resistant colonies were screened for recombinants. The Ad4 recombinants were confirmed by restriction digestion and midiprepped using the Qiagen Hi-Speed Midiprep kit. The recombinant Ad4-ΔE1-HA-con viruses were rescued by transfecting the PacI linearized genomes into 293 cells using the Polyfect reagent. Therefore, we constructed four Ad4 and four Ad5 vaccines that expressed each of the H1, H2, H3 and H5 consensus genes. All adenoviruses were purified using two sequential CsCl ultracentrifugation gradients and virus particles (vp) were quantitated by OD260.

### Ad Vaccine Immunization

The mice were anesthetized i.p. with ketamine (140 mg/kg)/xylazine (5.55 mg/kg). In order to create the multivalent vaccine we combined the Ad4 viral vectors expressing the H1, H2, H3 and H5 consensus antigens together. The 1 × 10^7^ vaccine dose contained the following viral vectors: 1 × 10^7^ vp of Ad4-H1-con, 1 × 10^7^ vp of Ad4-H2-con, 1 × 10^7^ vp of Ad4-H3-con and 1 × 10^7^ vp of Ad4-H5-con. Therefore, the 1 × 10^7^ vp/mouse dose contained 1 × 10^7^ vp of each Ad4-HA-con vaccine for a total of 4 × 10^7^ vp/immunization. The 5 × 10^7^ vaccine dose contained the following: 5 × 10^7^ vp of Ad4-H1-con, 5 × 10^7^ vp of Ad4-H2-con, 5 × 10^7^ vp of Ad4-H3-con and 5 × 10^7^ vp of Ad4-H5-con. Therefore, the 5 × 10^7^ vp/mouse dose contained 5 × 10^7^ vp of each Ad4-HA-con vaccine for a total of 2 × 10^8^ vp/immunization. The 1 × 10^10^ vaccine dose contained the following: 1 × 10^10^ vp of Ad4-H1-con, 1 × 10^10^ vp of Ad4-H2-con, 1 × 10^10^ vp of Ad4-H3-con and 1 × 10^10^ vp of Ad4-H5-con. Therefore, the 1 × 10^10^ vp/mouse dose contained 1 × 10^10^ vp of each Ad4-HA-con vaccine for a total of 4 × 10^10^ vp/immunization. The mice were boosted at 4 weeks using the Ad5-HA-con vaccines in a similar manner as the Ad4 vaccine formulation. The Ad vaccines were delivered intramuscularly (i.m.) in a total volume of 50 μl delivered in two 25 μl injections in the quadriceps. Two weeks after boosting, submandibular test bleeds were performed and sera was separated using Becton Dickinson serum separator tubes. The mice were challenged with lethal influenza virus at 4 weeks post-boosting.

### FluMist and FluZone Vaccines

The traditional inactivated, FluZone, 2010–2011 Formula (NR31063), and live-attenuated, FluMist, 2010–2011 Formula (NR-21987), vaccines were obtained from the BEI repository. FluZone is a split-virus vaccine prepared from influenza viruses A/California/07/2009 X-179A (H1N1)pdm09, A/Victoria/210/2009 X-187 (an A/Perth/16/2009-like virus) (H3N2) and B/Brisbane/60/2008^40^. FluMist is a live, trivalent vaccine that produces the hemagglutinin and neuraminidase surface antigens from reassortant influenza viruses A/California/07/2009 (H1N1)pdm09, A/Perth/16/2009 (H3N2) and B/Brisbane/60/2008 that were predicted to circulate in the United States during the 2010 to 2011 influenza season^40^. BALB/c mice were immunized with a dose of each vaccine that was equivalent to 10X a human equivalent dose. Mice received the FluZone vaccine (25 ng of each HA protein/mouse) intramuscularly in a total volume of 50 μl delivered in two 25 μl injections in the quadriceps. Mice were anesthetized with ketamine/xylazine as previously described and vaccinated intranasally with FluMist. The mice were placed on their back and 10^3^–10^4^ fluorescent focus units of each attenuated virus in a total volume of 4 μl (2 μl/nare) was pipetted into the nares and rested for 5 min. to allow for vaccine adsorption. Four weeks after immunization the mice were boosted with a second dose of FluZone or FluMist. Submandibular bleeds were collected at 2 weeks post-boosting and the mice were challenged with lethal doses of influenza at 4 weeks post-boosting.

### Influenza Viruses

The following influenza viruses were obtained from ATCC or the Biodefense and Emerging Infectious Diseases Repository: Influenza virus H1N1 A/PR/8/34 (PR/34), [ATCC-VR95], H1N1 A/Fort Monmouth/1/47 (FM/47) [BEI NR-3170], H1N1 A/WS/33 (WS/33) [BEI-2759], H1N1 A/California/07/09 (CA/09) [BEI-NR13663], H3N1 A/Texas/1/77 (TX/77) [BEI NR-3604], H3N2 A/Mississippi/1/85 (MS/85) [BEI NR-3502], H3N2 A/Aichi/2/68 (Aichi/68) [BEI NR-3483], and H3N2 A/Brisbane/10/2007 (Bris/07) [BEI NR-12283]. The following influenza viruses were received from the WHO GISRS network: H5N1 A/BH Goose/Qinghai/A/05 (Goose/05), H5N1 A/Vietnam/1203/04 (Viet/04), and H5N1 A/Japanese White Eye/Hong Kong/1038/2006 (White Eye/06). The mouse-adapted H3 and H5 influenza viruses were obtained through serial lung passaging in mice as previously described^41^. All of the viruses were passaged one time in SPF embryonated eggs and the chorioallantoic fluid was stored at −80**°**C. The influenza virus stocks were titered in BALB/c mice to determine the 50% mouse lethal dose (MLD_50_). No select agents were used in any of these studies and all virus and animal research was performed under approved BSL2 conditions.

### Animals

Female BALB/c mice (6–8 weeks old) were purchased from Charles River Laboratories (Wilmington, Massachusetts, USA) and housed in the University of Nebraska, Lincoln (UNL) Life Sciences Annex under the Association for Assessment and Accreditation of Laboratory Animal Care (AALAC) guidelines with animal use protocols approved by the corresponding the UNL Institutional Animal Care and Use Committee (IACUC protocol No. 1217). All animal experiments were carried out according to the provisions of the Animal Welfare Act, PHS Animal Welfare Policy, the principles of the NIH Guide for the Care and Use of Laboratory Animals, and the policies and procedures of UNL Office of Research Institutional Animal Care Program (IACP).

### Hemaglutination Inhibition (HI) Assay

HI titers were performed as previously described^4^. Briefly, sera was collected using Becton Dickinson microtainer tubes with serum separator, treated with receptor destroying enzyme (Denka Seiken) at 37 **°**C for 2 hrs and heat-inactivated at 56 **°**C for 1 h. Starting at a dilution of 1:5 sera were diluted two-fold in 50 µl of DPBS in a 96-well, nonsterile, nontissue culture–treated, round bottom microtiter plate. Four HAU of influenza virus in 50 ul was added to the diluted sera and incubated at room temperature (RT) for 1 hr. After incubation, 50 µl of a 1% chicken RBC solution was added and incubated at RT for 1 hr. The HI titer was determined to be the highest serum dilution to inhibit hemagglutination.

### Neutralization Assay

Sera from mice immunized with FluZone, FluMist or the multivalent 1 × 10^10^ vaccine dose was collected using Becton Dickinson microtainer tubes with serum separator. The sera was diluted 1:10 in cDMEM with 5%FBS and then serially diluted 2-fold to a final dilution of 1:640 in a final volume of 50 µl in a 96 well U bottom tissue culture plate. 50 µl of cDMEM-5 containing 50 TCID_50_ of each virus was added to the diluted sera. The plate was incubated for 1 h at 37 **°**C. After incubation, 100 µl of cDMEM-5 containing 2.5 × 10^5^ MDCK cells was added. The plate was incubated 24 h at 35 **°**C with 5% CO_2_. The media was removed using a multichannel pipettor and replaced with DMEM containing streptomicin (100 µg/ml), penicillin (100 µg/ml) and 2 µg/ml of TPCK-treated trypsin. The plate was incubated for 4 days at 35 **°**C with 5% CO_2_. 50 µl of 0.5% chicken RBC solution was added to all wells and incubated at room temperature for 1 h before reading. The neutralization titer was determined as the highest dilution of sera to prevent agglutination.

### Tissue Culture Infectious Dose (TCID_50_) and AdenoX Rapid Titer

In order to measure the infectivity of the viral vectored vaccines we performed a traditional TCID_50_ assay and a novel AdenoX rapid titer assay. The TCID_50_ was performed by serially diluting the Ad4 and Ad5 viruses in cDMEM containing 10% FBS in a final volume of 100 µl. Then 100 µl of cDMEM-10 containing 2.5 × 10^5^ 293 cells was added to all wells. The plates were incubated 7 days at 37 **°**C with 5% CO_2_ and 100 µl of fresh cDMEM-10 was added. The plates were read by microscope at days 10–14 and the TCID_50_ was determined by the highest dilution to show CPE. The AdenoX rapid titer was performed as described in the kit instructions. Briefly, 293 cells are infected with the Ad vectors for 48 hours at 37 **°**C with 5% CO_2_. The 293 cells are then fixed with methanol and stained with an anti-hexon monoclonal antibody. The infected cells are counted and infectious particles are determined by calculating hexon-positive cells/surface area.

### Lethal Influenza Challenge

The mice were subjected to a stringent lethal challenge four weeks after the booster immunization. The lethal virus was administered intranasally into mice that were anesthetized i.p. with ketamine (140 mg/kg)/xylazine (5.55 mg/kg). The mice were placed in a supine position and 20 µl of influenza virus was pipetted into the nares in two 10 µl volumes. Baseline weight measurements were determined prior to the lethal influenza challenge. The vaccinated and control mice received the following influenza challenges: 100 MLD_50_ of A/PR/8/34 (H1N1), A/FM/1/47 (H1N1), A/WS/33 (H1N1), A/TX/1/77 (H3N1), A/Vietnam/04 (H5N1), A/BH Goose/Qinghai/A/05 (H5N1), A/Japanese White Eye/Hong Kong/1038/2006 (H5N1). Mice that were challenged with A/Mississippi/1/85 (H3N2) and A/Aichi/2/68 (H3N2) received a 10 MLD_50_ dose of influenza virus. Body weight and signs of disease were observed daily. Mice were humanely euthanized if their body weight dropped ≥25% of baseline weights.

### Peptide Microarray

We screened sera from mice immunized with FluZone, FluMist or the multivalent 1 × 10^10^ vaccine dose in order to determine which specific linear antibody epitopes were induced by the Ad-vectored HA vaccine. Sera from each group was pooled and used to screen a custom peptide microarray. The PEPperCHIP® Peptide Microarrays were produced by B-Bridge International (Cupertino, CA) using a Poly(ethylene glycol)-based graft copolymer coating with a thickness of 13,5 nm and an additional three amino acid linker (ß-alanine, aspartic acid, ß-alanine) to each peptide. The microarray consisted of four HA antigens (H3-con, A/Aichi/2/1968, A/Texas/1/1977 and A/Mississippi/1/85_partial) were linked together and then translated into 2,040 different overlapping 15 aa peptides printed in duplicate (4,080 peptide spots each array copy). Each PEPperCHIP® Peptide Microarray contains two array copies (see layout scheme below). At each array copy Flag (as DYKDD-fragment) and HA (as DVPDYA-fragment) was incorporated at the beginning resp. at the end of the array as control peptides (2 spots each control peptide).

The microarrays were probed using sera from immunized transgenic mice as described in the PepperCHIP immunoassay protocol^42^. Briefly, the microarray was blocked with PBS, pH 7.4/0.05% Tween 20/1% BSA for 30 min. at room temperature. The array was washed 3 times with standard buffer (PBS, pH 7.4/0.05% Tween 20) and incubated with sera diluted 1:1000 in standard buffer + 0.1% BSA for 1 hour. The sera were removed and the array was washed 3 times for 5 minutes using standard buffer. Bound mouse sera was detected using Cy3 Goat anti-mouse IgG (H + L) antibody (Life Technologies, Eugene, OR). The array was washed as previously described and dried for scanning. Control peptides were detected using standard anti-HA and anti-FLAG DyLight 680 and 800 antibodies as previously described^42^. All incubations were performed on a shaking platform at 140 RPM. The microarrays were scanned at 5 uM resolution using the Axon GenePix 4000B scanner.

### ELISPOT Assay

The ELISPOT assay was performed as previously described^24^. Briefly, splenocytes were prepared from immunized mice using a 40-µm cell strainer and the red blood cells were lysed using and ACK lysis buffer. The splenocytes were washed and resuspended in cRPMI-10% FBS. The splenocytes from groups of 5 immunized mice were pooled. The splenocytes were mixed with peptide pools and added to 96 well Immunlon-P filter plates (Millipore) that had been coated with anti-mouse interferon-γ AN18 antibody (MABTECH). The plates were incubated at 37 **°**C with 5% CO_2_ overnight. The plates were washed and mouse interferon-γ was detected using R4-6A2 antibody and streptavidin-ALP (MABTECH). The spots were developed using BCIP/NBT substrate (Moss). The peptides consisted of 17-mers overlapping by 12 amino acids (Genscript). A total of 258 peptides were included in the pools and the sequences represented included, A/TX/1/77, A/Aichi/2/68, A/Mississippi/1/85 and the H3-con protein.

### Western Blot

293 cells were infected with 500 viral particles/cell, and incubated overnight at normal conditions. Samples were collected and diluted 1:1 with 2X SDS lysis buffer containing 5% β-mercaptoethanol (BME) and boiled for 10 minutes prior to loading. Samples were loaded onto an 8% SDS-PAGE gel to separate the proteins, following the standard electrophoresis procedure. Separated proteins were transferred to a PVDF membrane at 65 V for one hour. Western blots were conducted for detection of H1, H2, H3, and H5 proteins. PVDF membranes were blocked 30 minutes in a 5% non-fat dry milk in 1X TBST solution. PVDF membranes were incubated in primary antibodies (purchased from BEI resources) overnight at 4 °C in 1X TBST + 1% milk at the following dilutions: 1:1000 for goat α-H0 serum(A/PR/8/34), 1:1000 for goat α-H2 serum(A/Singapore/1/57), 1:1000 for goat α-H3 serum(A/Aichi/2/68), and 1:1000 for goat α-HAV5 serum(A/Tern/S. Africa/61). Membranes were washed three times, two minutes each, in 1X TBST buffer. PVDF membranes were incubated 30 minutes at room temperature in a 1:2000 α-goat IgG HRP conjugated dilution in 1XTBST + 1% dry milk for the secondary antibody. Membranes were washed three times, two minutes each, in 1X TBST buffer. Membranes were developed using SuperSignal West Pico Chemiluminescent Substrate following the manufacturer’s instructions. Blots were imaged with the BioRad ChemiDoc MP imaging system. Due to differences in the goat polyclonal affinities the blots were developed using various lengths of exposure (Fig. 8A). In order to identify differences in overall protein detection we compared the ability of the polyclonal primary antibodies to detect the wildtype influenza virus genes. Ad-HA-con samples were prepared as previously described and compared to 1.5 hemagluttinating units of each wildtype influenza that was resuspended in laemmli buffer, and boiled for 10 minutes. All of the proteins were run on the same 12.5% SDS-PAGE and transferred to a PVDF membrane for blotting. The blocked membrane was probed with a 1:2000 dilution of the polyclonal antibodies previously described for H1, H3 and H5. The blot was probed with the secondary α-goat IgG HRP for 1 hr at a dilution of 1:2000. The blot was washed, developed using SuperSignal West Pico Chemiluminescent Substrate and imaged with the BioRad ChemiDoc MP imaging system (Fig. 8B).

### Data availability statement

The datasets generated during and/or analyzed during the current study are available from the corresponding author on reasonable request.

## Electronic supplementary material

Supplementary Figures
