# Supplementary material for: Efficacy of an Adenoviral Vectored Multivalent Centralized Influenza Vaccine
Source: Sci Rep. 2017 Nov 2;7:14912. doi: 10.1038/s41598-017-14891-y (PMC5668234; doi:10.1038/s41598-017-14891-y)

Supplementary Figures:

# Efficacy of an Adenoviral Vectored Multivalent Centralized Influenza Vaccine

Short Title: Establishing a Foundation of Influenza Immunity.

Amy Lingel, Brianna L. Bullard and Eric A. Weaver*

*Supplemental Figure 1. Construction of Ad5-HA viral vectors.*

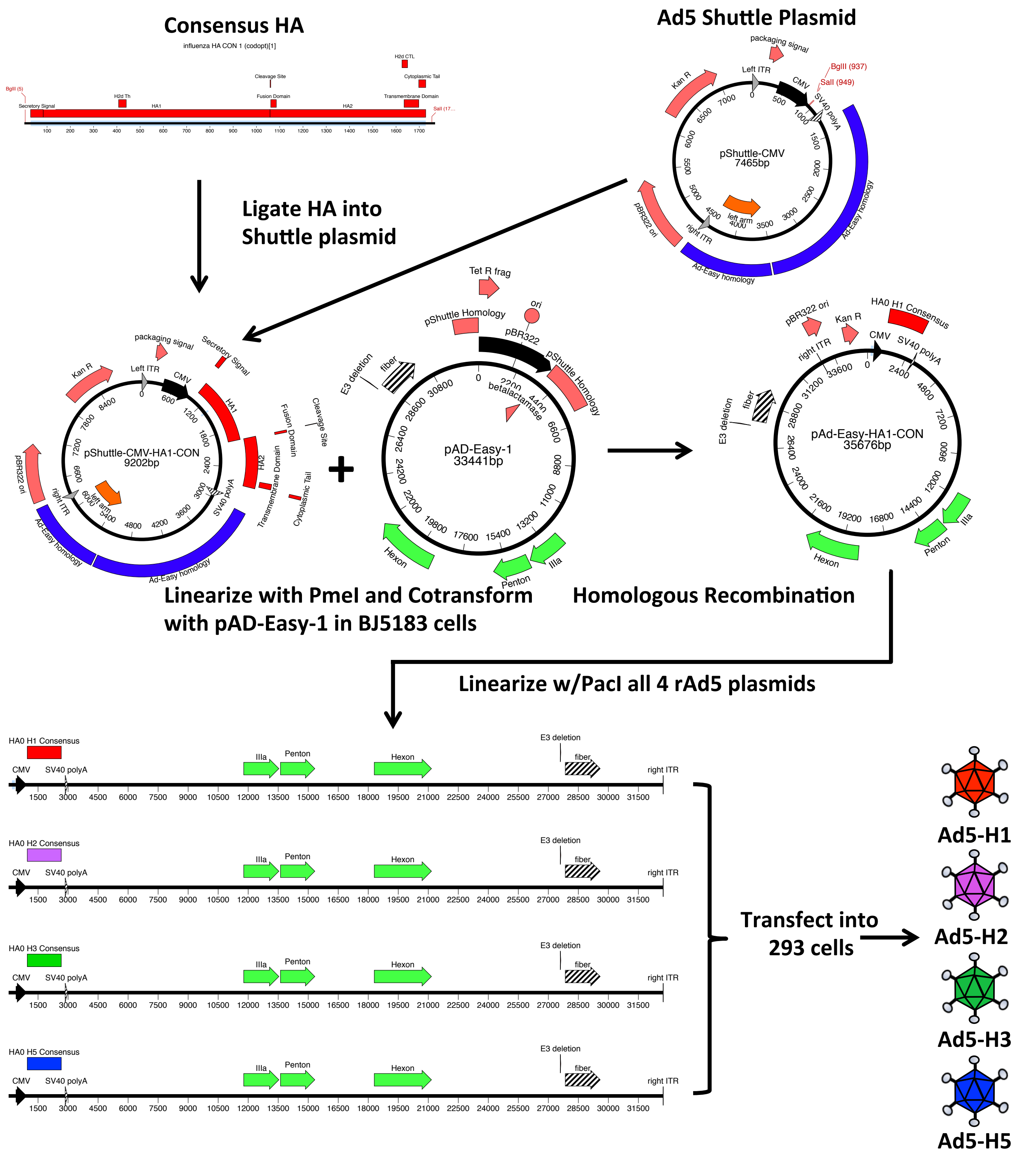

*Supplemental Figure 2. Construction of Ad4-HA viral vectors.*

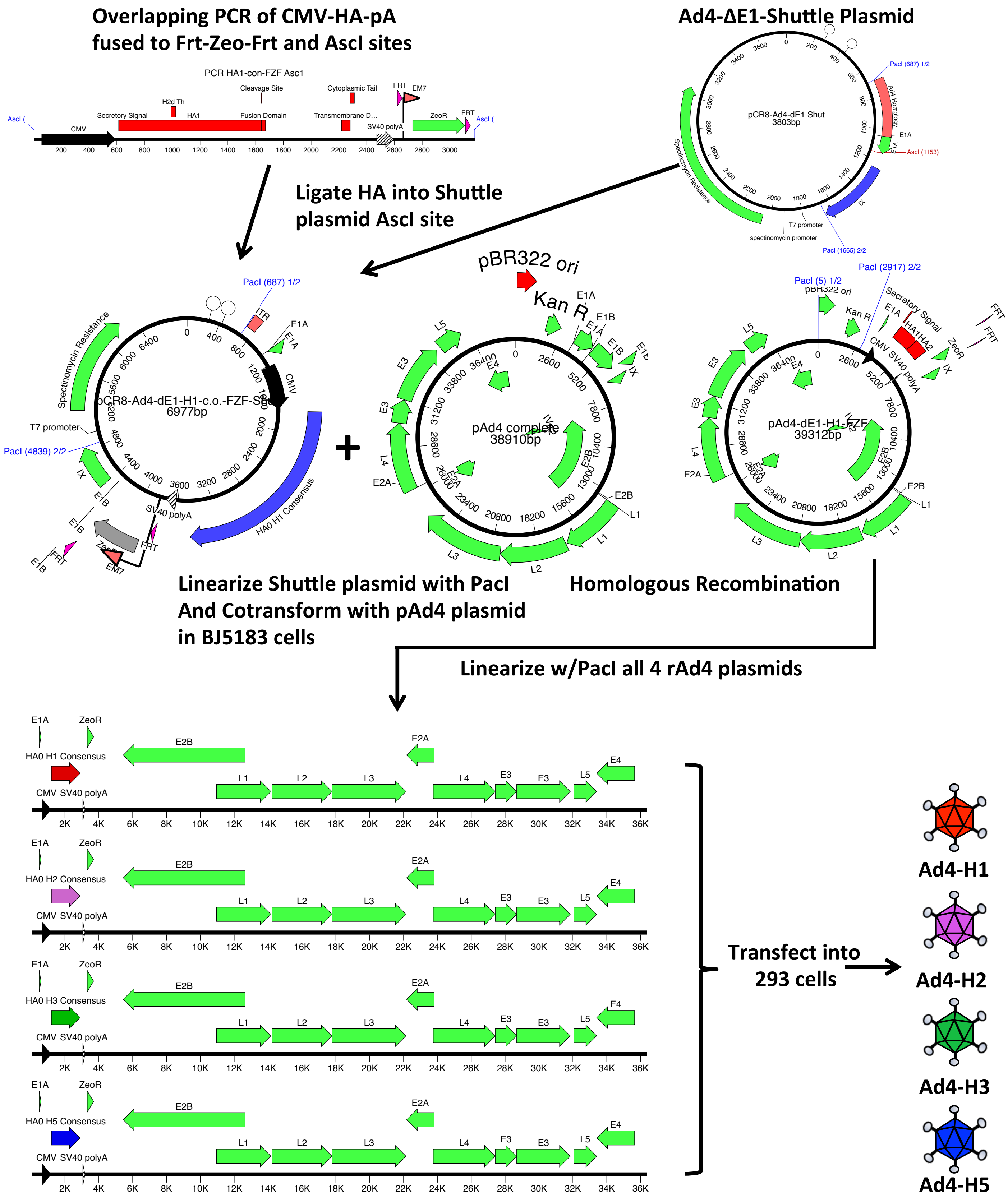

Supplement: Supplementary file 1 — Supplementary Figures [file 41598_2017_14891_MOESM1_ESM.pdf]
